# The Hospital. Nursing Section

**Published:** 1904-07-02

**Authors:** 


### The Hospital.

### Nursing Section.

Contributions for this Section of "The Hospital" should be addressed to the Editor, "The Hospital" Nursing Section, 28 & 29 Southampton Street, Strand, London. W.C.

No. 927.—Vol. XXXVI. *SATURDAY, JULY 2, 1904.*

### Notes on News from the Nursing World.

### THE DUCHESS OF ALBANY AND THE KINGSTON INFIRMARY NURSES.

On the occasion of the visit of the Duchess of Albany last week to Kingston Infirmary, her Royal Highness afforded the nurses considerable gratification. On her arrival at the infirmary she was received by a number of officials, including the assistant matron, Miss A. Smith, and a number of nurses were also waiting at the entrance to assist in giving her a warm welcome. The Duchess having inscribed her name in the visitors' book, was presented by Miss Smith, on behalf of the nurses, with a lovely basket of roses and orchids. She was then conducted round the wards by the Rev. F. O. Sutton and Dr. Donald. She spent upwards of two hours in the infirmary, and passed from bed to bed, speaking kindly words of cheer and sympathy to every patient. She expressed herself as much pleased with all she saw. Before leaving the infirmary the Duchess took tea on the lawn, and was afterwards photographed in a group with the nurses who were present.

### THE NURSES' HOME AT ST. THOMAS'S HOSPITAL.

Mr. J. G. Wainwright, treasurer of St. Thomas's Hospital, in his speech on the occasion of the prize-giving to successful medical students last week, drew attention to the new building at the corner of the block facing Westminster Bridge Road. This is the new home for the nurses, which has already reached the third floor. Nothing, said Mr. Wainwright, showed how the services of nurses had gained public recognition more than the fact that, when the hospital was opened 95 was considered the largest number that could ever be required, while, after 33 years' work, the staff of 176 urgently required to be brought up to 216, or even 226. The ground and first floor of the building will be for the patients of St. Thomas's Home, and 185 nurses will be accommodated in this and the adjoining block.

### NURSING IN A NORWEGIAN INFIRMARY.

An excellent idea is given of the nursing in a Norwegian infirmary, in our columns this week, by an English nurse who has paid a visit to Bergen. She found that of the two head nurses at the General Infirmary, one was trained in Germany, and the other at the Edinburgh Royal Infirmary. The training at Bergen, it will be seen, is on much the same lines as that in English hospitals, but amongst other differences, the early hour at which operations begin—namely, 8 a.m.—will be noticed. The absence of old chronic cases is attributed by our contributor to the number of homes for the old and infirm in Bergen. The description of the "pride of the infirmary" will be read with envy by many nurses in English institutions. It is a splendid bath-house, in charge of trained masseurs, male and female.

### THE SELECT COMMITTEE ON REGISTRATION.

At the instance of Sir A. Acland-Hood, chief Government Whip, a Select Committee was appointed on Friday to consider the expediency of providing for the registration of nurses. The members nominated to serve are :—Dr. Ambrose, Major Bagot, Mr. Henderson, Mr. Lyell, Mr. Malcolm, Lord Morpeth, Mr. Pierpoint, Sir J. Stirling-Maxwell, Mr. Tennant, and Sir J. Batty Tuke. It will be observed that neither Dr. Farquharson nor Mr. Claud Hay, who are, or were, in charge of the rival bills, is on the Select Committee.

### THE NURSES' HOME AT HEATHERSIDE.

After the opening by the Prince and Princess of Wales of the new sanatorium attached to Brompton Consumption Hospital, at Frimley, on Saturday, an inspection was made of the house which will be occupied by the nursing staff. This is entirely separated from the main block, and is connected with the accommodation for the medical staff, by a glass-covered passage. The building consists of three storeys, and all the windows have a charming view of pine woods of the Surrey hills. On the entrance floor are the dining-room, coloured with cheerful red, and simply furnished in dark wood ; a sitting-room, with blue-green walls, dark blue carpet and sofas, and cosy chintz-covered easy-chairs ; a small dining-room or office, and ample lavatory, cloak-room and cupboard arrangements. The kitchens are also on this floor. On the two upper storeys are the bedrooms, all blue and white, with brown floor cloth and holland-coloured curtains, these being used in the sitting-rooms also with a very pleasing effect. The staircases and passages are coloured buff, and there are large cupboards on each landing. Each floor has its bath-room and lavatory, and there is provision for electric light throughout the house. Precaution against fire is provided by means of a break glass in the hall. The staff will consist of about 12 nurses with a lady superintendent. Some of the Brompton sisters and nurses were among those who travelled to Camberley by special train to witness the opening ceremony.

### THE POSITION OF THE POOR LAW NURSING QUESTION.

At the annual meeting of the National Association of Workhouse Masters and Matrons last week, a report concerning the present position of nursing, consequent upon questions which have recently been asked in the House of Commons with regard to the contemplated action of the Local Government Board upon the report of the Departmental Committee, was submitted. These questions have already been mentioned in our columns. The Council regard Mr. Long's promise to consider the suggestion that a draft of the new nursing order shall be submitted to them as satisfactory, but they intimate that if it contains regulations adverse to the position taken up by the Association representatives of Boards of Guardians in England and Wales, it will not go "unchallenged by high authority." The report was adopted.

### THE AMENDE HONORABLE.

It is always pleasant to have to record a frank recognition of a mistake in criticism. In the *Lady's Pictorial* for June 4th there appeared, under the heading of "Boudoir Gossip," a paragraph suggesting that the report of the Royal National Pension Fund for Nurses would give the enemy cause to blaspheme, "inasmuch as it practically states that there is probably no work in which women engage which more surely brings them to the 'hymeneal altar' than that of nursing." Two weeks later, under the heading of "A Deserving Fund," the editor of our contemporary having evidently, in the interval, carefully perused the report which his contributor could not have read, devoted a short article to the task of showing that facts and figures prove that the "fine band of women workers" attached to the Fund do not "in any way seem to make matrimony the goal of their ambition." The report itself, our contemporary adds, indicates that "the majority of nurses do not marry and do work whole-heartedly at their arduous calling"; and, seeing that only 160 members of the Fund married and gave up nursing during the past year, while nearly 1,000 new policies were issued, there can be no room for doubt in the matter. Of course, our readers do not require to be told that the experience of the managers of the Pension Fund is that the overwhelming majority of the members need the provision they are able, through its agency, to make for themselves, but we are glad that our contemporary, having published an erroneous conclusion which was not altogether complimentary to the Fund, has offered the most handsome reparation by pointing out its true purpose.

### DEATH OF A VOLUNTEER NURSE IN NEW GUINEA.

It will be recollected that in our issue of April 30 we published an account of the nursing at the Anglican Mission Station, River Mambare, British New Guinea, in which the writer alluded to the need of more helpers. We regret to learn from Bishop Stone-Wigg that this need has been accentuated by the death at Hioge, of Nurse M. Alicia Newton, the first English lady volunteer who has passed away in the diocese.

### DISTRICT NURSING IN GLASGOW.

At the ninth annual meeting of the subscribers to the Maryhill District Nursing Association, the report was adopted. The two nurses employed by the Association work in the 'Maryhill' Ward of the City of Glasgow, and last year they paid 846 more visits than in the previous year, attended nine more patients, and devoted some 237½ more hours to their duties. It is an encouraging feature that the voluntary thankofferings received from patients or their friends, were greater in both number and amount than on any former occasion. The report gives the subscriptions in a tabulated form, and shows exactly what the collector in each district has done, with the names and addresses of every one of the 2,436 subscribers. After allowing for a contribution of ten guineas to the parent association, a balance of £28 has been carried to capital account. We congratulate the committee on the admirable manner in which the organisation is managed.

### FLOWER CARNIVAL AT WESTHOUGHTON.

The Ladies' Committee of the Westhoughton Sick Nursing Association conceived the happy thought of organising a flower carnival in aid of the funds. The event included the crowning of a May Queen, which was none the less appreciated because the ceremony occurred in June. No fewer than 2,300 children of both sexes took part in the demonstration, of whom 500 wore daffodils, 200 poppies, 200 chrysanthemums, 150 tulips, 200 roses, 60 dahlias, 200 primroses, 200 purple iris, and 300 marguerites. The fact that the flowers were artificial did not detract from the general effect, and when grouped in their respective sets the colour schemes formed by the children were exceedingly vivid. The principal streets having been paraded the procession made their way to a field, in which the crowning ceremony was performed and a programme of sports was carried out. Nearly 3,000 persons paid for admission and the amount cleared, after payment of all expenses, reached the respectable total of over £50.

### KENT AND CANTERBURY INSTITUTE FOR TRAINED NURSES.

On Monday afternoon about 100 guests assembled in the garden of the Kent and Canterbury Institute for Trained Nurses, on the invitation of the House Committee, to witness the presentation of testimonials to Miss Shaw, late Lady Superintendent, and Miss Alice Pereira, of the Kent and Canterbury Nurses' Institute. Miss Shaw retired about three months ago, owing to failing health, from the post of lady superintendent, which she held with much credit for the past 12 years. When she first took over charge there were only eight nurses, and the institute was in an early stage of rather doubtful struggling. There are now 43 nurses, and the institute has an assured reputation all over Kent. It was felt that Miss Shaw's many friends in Canterbury and neighbourhood would like to present her with a testimonial. A sub-committee undertook the arrangement. Subscriptions were received from 51 subscribers, and it was decided that the testimonial should take the form of a piano. Nurse Alice Pereira has served the institute well for the past 12 years, and under the rules has become entitled to a gratuity of £10. The Bishop of Dover, in making the presentation, congratulated Miss Shaw on her successful career, expressed the Committee's regret at losing her services, and the pleasure of her many friends in presenting this mark of their regard. He could not hand over the piano because it has not yet been purchased, owing to the fact that Miss Shaw intends to spend another winter abroad for the complete restoration of her health before settling down in an English home; but he gave symbolical possession by handing to her a silver plate, which will eventually be fixed to the piano, and an illuminated list of the names of the subscribers. The Bishop took the opportunity of welcoming the new lady superintendent, Miss Steen, and proceeded to present the cheque for £10 to Nurse Alice Pereira, congratulating her and the nurses generally on the good work they are doing and the general esteem in which they are held. After the presentations Miss Steen and the nurses entertained their guests at tea.

### BICYCLE ACCOMMODATION FOR NURSES.

It having been stated at a meeting of the Edinburgh Town Council that the Public Health Committee recommended the provision of bicycle accommodation for the nurses of the City Hospital, at a probable cost of £60, the Lord Provost said that "it seemed a funny thing to ask for £60 for a nursery for bicycles." We do not quite see the comic aspect. If the nurses of the Edinburgh City Hospitals are to be permitted to keep bicycles they must obviously have a place to put them in. In this instance the place is there, and it is only necessary to cover in with glass the sides of the corridor from the servants' to the nurses' home. The recommendation was ultimately agreed to, in spite of the fact that the Lord Provost declared that he could put up a house with a corrugated roof for £25. In ordinary circumstances £60 seems a good deal for glazing, but we conclude that the conditions are special.

### USEFUL BOOKS FOR NORWICH NURSES.

On the recommendation of the house committee the Norwich Board of Guardians have agreed to the purchase of a number of books on nursing, medicine, and kindred subjects, for the library of the nurses' home. This is a step in the right direction, and we hope that it will be emulated by other boards. The cost of a few useful books is very small, and the practical advantages to be gained by nurses from studying them considerable. We do not suggest that they should spend all their leisure in reading works on nursing and medicine, but in the absence of such works there is a natural tendency to spend too much time in the perusal of fiction, which, however interesting or elevating, cannot supply their place.

### DEVELOPMENT OF WORK AT HOLYWOOD.

Like the Cathedral Nursing Society of Newcastle-on-Tyne, the Holywood Nursing Society has come of age, and though the latter organisation is only, in comparison, a small one, the report and the speeches delivered at the annual meeting show that it is in a thoroughly healthy condition. During the year just concluded the number of cases nursed was 193, and the number of visits paid 4,469. In the previous year the Committee were able to invest money in Stock; last year, as the report says, they laid it out to more advantage by securing the services of a maternity nurse. The latter investment was so speedily and highly appreciated that it soon became necessary to procure a bicycle to ensure all patients being attended. As to the financial position, there was a credit balance of £12 18s. 2d., and the gratifying announcement was made that the Committee now feel justified in proceeding with the erection of a nurses' home. Perhaps one reason why the Holywood Nursing Society, which is affiliated to Queen Victoria's Jubilee Institute, is so prosperous is that the clergy and ministers of all denominations work to promote its success. Seven of them spoke at the meeting, and Canon Moore said that it seemed to him that the Association brought into prominence in a practical way the essential spirit of true Christianity.

### SALFORD GUARDIANS AND THEIR NURSES.

A short time ago three of the head nurses at Hope Hospital, Salford, tendered their resignations to the Guardians because the latter had allowed the complaints of certain patients to be made public. The Infirmary Committee have, however, since met, and, after discussing the question, they passed a vote of confidence in the three nurses and decided to ask them to withdraw their resignations. A protest from the probationers in the hospital against the publication of the complaints was also considered, and the Committee resolved that it should lie on the table. It may, therefore, be concluded that the accusations against the nurses were found to be without foundation.

### HEREFORDSHIRE HOSPITAL AND PRIVATE NURSING.

At the last meeting of the Board of Management of Herefordshire General Hospital it was decided to appoint a lady dispenser, and a Miss M. C. Nichol, of Westminster General Dispensary, was selected for the post, with a salary of £80 a year, luncheon and tea. The report of the Committee as to the future disposition of the private nursing fund was then presented, but a decision respecting it was postponed, and it was ordered that the report should be printed and circulated. One of the most important of the suggestions of the Committee deals with the question of payment, and is to the effect that so long as the number of private nurses does not exceed twenty a contribution shall be made by the nursing fund to the hospital of £150 per annum. This contribution is to cover rent, rates, firing, lighting, servants' wages, and remuneration to the matron. It seems a reasonable amount, and should form the basis of an arrangement satisfactory to all parties.

### ANNEXING THE NURSES' GARDEN.

A Bristol correspondent calls attention to the fact that at the back of the Nurses' Home connected with the Workhouse Infirmary at Stapleton the guardians are erecting huts for phthisical women. The huts, our correspondent states, will be within a few feet of the sleeping apartments of the nurses, and they are being put up on the only piece of garden available. The nurses are thus simultaneously to lose their garden and to have the phthisical patients placed in close proximity to their bedrooms. In the interests of the guardians themselves, as well as of the nurses, we hope that it is not too late to reconsider an arrangement which, unless indispensable, should not be persisted in if only because it may add to the difficulties of filling up vacancies in the nursing staff.

### THE HEALTH OF THE EDITOR.

In reply to a great number of kind inquiries which have been received by the Secretary of the Royal National Pension Fund for Nurses, and by ourselves, respecting the health of the Editor, we are glad to state that Sir Henry Burdett is making satisfactory progress towards recovery.

### The Nursing Outlook.

"From magnanimity, all fear above;
From nobler recompense, above applause,
Which owes to man's short outlook all its charm."

### RED TAPE IN THE WARDS.

Everyone knows in theory that the difference between a good nurse and a bad one is that the former considers her patient first, and the second considers herself first. As a sarcastic visitor once put it, on noting the immaculate gowns of the nurses and the spotlessness of the ward: "Obviously a nurse's first duty here is to herself, her second to the ward, and her third (if she has time) to the patients." This same observer discovered many of the details of ward life which seem to remain even unknown to the authorities, and which tend to support her maxims. For instance, in one large hospital it is the custom to skim the cream off the milk provided for the patients, and use it for the sister's tea! For sheer dishonesty and unmitigated selfishness this is hard to beat, and it is no wonder that probationers trained in such deeds should prove utter failures when they are sent out to private nursing, where the patient is of some importance. Another detail culled from another hospital is, that in the surgical wards it is usual to turn the blankets back at the foot instead of tucking them in, so that the opening up of the bed for the surgeon and dressers can be more easily performed. A patient complained of the draught to his feet and the unaccustomed cold, and was promptly reported to the sister for grumbling. Since then every patient who complains is warned by his neighbours that he must submit to this utterly unnecessary discomfort, just because it keeps the beds tidier for the nurses.

Here is another little story of lack of consideration where detail is concerned. In giving cod-liver oil in one hospital in London it is customary to use an egg-cup, in order to avoid tainting the common medicine glass. This cup is as a rule kept in the patient's locker, and is not washed between doses, with the result that the oil gets a rancid taste which is extremely objectionable to the patients, though they dare not complain. We trust that the matrons and sisters of London hospitals will look in a few lockers and see if this tale refers to their domain. One sarcastic inspector also warns us never to look down the spout of a feeding cup; she believes that the spout is never washed, from the brown, furred condition in which she has usually found it, and she wonders what milk and beef-tea must taste like when drunk through that foul funnel, but she has not the courage to try.

Then it is on record that a patient in a city hospital once caused a frightful uproar by taking in a tooth-brush and asking the nurse to supply him with warm water to wash his teeth. There was fine indignation: what were these patients coming to next? Fancy expecting the nurse to wait on him as though she were a valet!

Again, in one hospital the probationers are trained to remove all pillows from the bed when changing the sheets in a helpless case. It is very little more trouble to gently remove the under pillow, take it away, and give it a good shake, and put it back on the freshly-prepared side of the bed, and it is much more comfortable not to leave the patient's head quite flat. Imagine the indignation of the private patient if this were done to him and he was well enough to protest! Obviously it is this want of consideration for the patient which is taught in too many of the hospitals that is at the bottom of the unpopularity of the private nurse—taught not in theory, but in practice, taught by example, by the daily routine and the daily hurry. It is all very well to teach nurses to be "smart" in their work; but the continual "Hurry up, nurse!" "Pull out that sheet!" "You have been 10 minutes over that bed!" does not conduce to gentleness of manner and tenderness of touch. And then, in private nursing, where there is no such rush, the nurse still goes at her work in the same slap-dash manner, and thinks that if the counterpane does not hang with mathematical precision the doctor will consider her badly trained.

And how aghast this machine-made nurse looks when a private patient asks not only the way to clean her own teeth, but requests the nurse to clean her false ones for her! Yet the majority, perhaps, of adult patients who pay are possessed of false teeth, and it is obviously the private nurse's duty to know the proper method of cleaning them—holding them in a napkin the while, and handing them back when done in a glass bowl of tepid water.

One incident which came to our knowledge lately was laughable, and yet very characteristic. A private nurse had had charge of a pneumonia case in a West-end house, and the crisis was passed swiftly and safely, the patient two days later, though weak, being quite herself again. She became conscious of the two plaits of hair hanging one over each shoulder, and asked somewhat anxiously for a looking-glass. When she beheld her neatly-parted and smoothly-brushed hair she burst into tears—"A Dutch doll!" she cried; "I look like a Dutch doll, and everyone has seen me like that!"

Of course, most nurses find their hearts again when once free from the deadening routine of hospital work, and somewhat shame-facedly learn to sympathise with their private cases and to behave like thinking, feeling human beings again. But is it not worth while for those in authority in the hospital wards to inquire into a few of the details we have dealt with above? We have made general statements before on this question: now we have given actual facts culled from different hospitals, and we hope that those whom the caps fit will try and cure the spirit which leads to such inconsiderate treatment of hospital patients.

### Lectures Upon the Nursing of Mental Diseases.

By ROBERT JONES, M.D.Lond., B.S., F.R.C.S.Eng., M.R.C.P.Lond., Resident Physician and Superintendent of the London County Asylum, Claybury.

### LECTURE XIV.

THE trained mental nurse is expected to be familiar with the application of every method of remedial treatment. The use of (1) massage, (2) electricity, (3) physical drill, (4) hydro-therapeutics, and (5) the rest treatment are included in those measures which are now considered to be helpful in the cure of insanity.

As to (1) Massage, it is practically only a form of exercise, but exercise of a passive description, and applied to the patient by another person in contra-distinction to active exercise (such as dumbells or tennis) which is taken by the person himself. Massage is the name given to a number of manipulations of the body which are of great value in the treatment of nervous diseases. These movements are not difficult to learn, although intelligence is required in their application, and the *masseur* or *masseuse* must have his or her brains—so to speak—at that part of the hand which is used in the work, and the hand should neither be too small, so as to be inefficient in grasping or kneading the part treated, nor too large, so as to lose the fine sense and muscular touch necessary in the treatment of superficial and delicate structures such as nerves, veins, and lymph vessels. The movements are always made in the direction of the venous blood flow. The terms used in the description of massage are French, but their English equivalents are here preferred. There are four kinds of movements in the application of massage which must be carefully distinguished and apprehended, the whole value of massage being in the proper execution of these movements in their details. These movements are applied lightly or strongly, and superficially or deeply, and they are used either separately or in combination. They are generally commenced in the legs, then the arms, back, chest, and abdomen, and are as follows:—(*a*) *effleurage*, or gentle stroking, which consists in lightly passing the flat of the hand lightly over the skin of the limbs or body, always in the direction towards the middle of the body—*i.e.* towards the heart. The effect of such stroking is to empty the veins and the contents of the lymphatics, and thus relieve congestion.

(*b*) The next movement is *petrissage*, or kneading, and is used for the muscles of the limbs or the body, which are grasped with the whole hands, lifted, and kneaded, tightening and loosening these alternately. In the calf or thigh muscles both hands grasp the limb, and the muscles are rolled between the hands against the bone. There must be no pinching, and the skin must move with the hand, the same part of the skin rolling over the same part of the hand which does not slide over the skin and cause the fine hairs of the skin to be pulled.

(*c*) The third method is *massage à friction*, which is applied with the thumb, finger tips, or the whole hand in small circles, such as those where neuralgia has pained, or around the joints where effusions collect, or where there are fibrous or other swellings. This movement may consist of vigorous strokes with one hand, and strong circular or to-and-fro friction with the other.

(*d*) The last movement is described as *tapotement*, and is a striking or a percussion with the tips of the fingers, the ulnar edge of the hand or the whole open hand. This includes the application of gentle blows transversely across the long axis of a muscle and is not unlike "chopping" the muscle. It is applied with the finger-tips when stimulating a nerve-trunk, or with the open hand when applied to anæsthetic areas.

It is customary to apply massage direct to the skin and without the application of adventitious material such as oil, lanoline, or vaseline. It is also best to apply massage for a short time at first and once only in the day. When the patient becomes more accustomed to its use the application may, if desired, extend to an hour or more per day and may be applied twice. Care must be taken to cover over and keep warm the part massaged. In using massage for the abdomen, the head is raised upon a pillow, the knees are bent, and the patient breathes deeply. Deep friction is then used with the heel and palm of both hands in small circles round the umbilicus, over the liver and stomach, and particularly over the course and direction of the large intestine, the abdominal walls being well grasped and kneaded in the process.

For insomnia the neck is stroked downwards from the occiput or back of the head and on each side, thus aiding the flow of venous blood and lessening the amount of blood in the head, in this way favouring the anæmia which has been referred to as concomitant with sleep. For locomotor ataxia massage has been recommended as a valuable remedy and is said to lessen the pains and to cause the various affections and perversions of sensation to disappear. At any rate, it has the merit of doing something in the way of relief under conditions when everything else is of no avail. Nervous states described as neurasthenia, hysteria, muscular atrophy, and what are called occupation neuroses—such as writers' cramp, etc.—are definitely improved by massage. Speaking generally, massage increases the blood supply in the part rubbed. It causes the skin and the muscles to flush and the lymph stream to flow, thus removing fatigue-products and restoring functional activity to jaded organs. It certainly increases the activity of the superficial circulation, it increases the secretion of urine, and it has a definite influence upon nutrition. In melancholia, and conditions where the secretions or the excretions are out of order, and states such as sleeplessness, dizziness, confusion, migraine, headaches, and flushings, massage has been *found* to be very useful, for it improves the nutrition of the muscles when patients refuse or are unable to take exercise, or when they are sent out of doors and linger listlessly or sit indifferently about. It is well not to neglect the influence of electricity, full and free feeding, and due warmth in such cases, and means should be taken to record the weight weekly and to note the desire for food and the power to assimilate it. As to (2) Electricity, the nurse is *now* expected to be familiar with its application. It is of great advantage in certain forms of mental diseases; more especially those cases of early mental failure now more commonly seen than of yore among otherwise promising young men and women; also in cases of local paralysis, some of which may have been caused by lead-poisoning. Electricity improves the nutrition, as may be seen in the great improvement in health which occurs in these cases when undergoing treatment by electric baths. It is used for promoting the general tone, or that of individual muscles and nerves. It is also used to increase the sensibility of the skin, or as a sedative for the relief of pain and spasm. It is further used for cases of locomotor ataxia, or muscular paralysis and for sleeplessness. The laws which govern the flow of the electric current are similar to those which govern the flow of water. Water will flow from a higher to a lower level, and in doing this will exert force which is called its *potential*, viz., a capacity for doing work, and we see this potential made use of in turning water wheels which work machinery in mills and other factories. Electricity is described either as *positive* or *negative* in kind and the potential is higher in the positive than in the negative. The two kinds always tend to become united, that of higher potential tending to flow towards the lower. This tendency is the *electric force*. It is called the *electro-motive force*, is measured in *volts*, and always flows outside the battery from the positive to the negative. The amount of electricity which passes through a wire at any one time is measured in *ampères*, but in medicine we use only one-thousandth part of an ampère as our standard, and this is called a *milliampère*. Not more than from 5 to 15 milliampères are used medically. The body offers a certain amount of resistance to the passage of electricity, that is to say, it is not a good conductor of electricity. The standard of resistance is called an *Ohm*—after the name of a famous electrician who described it. The resistance of the body is much less when in water, generally speaking it may be described as from 3,000 to 5,000 Ohms. Electricity may be applied to the body in three ways, (*a*) *static*, or *electro-static*, that from a machine in which plates rub each other, the friction giving rise to electricity and used for nervous, hysterical or other pains such as those in neuralgia, headaches, in skin diseases or chronic ulcers, (*b*) that obtained by the direct chemical action of one substance on another, as when an acid dissolves a metal in a jar or cell, several cells making up what is called a *battery*, and the current the *battery current*. In using this current the negative pole is often glided over the muscles (labile electricity). The power of response of a paralysed muscle remains longest for the battery current than any other. The last variety is (*c*) that which is obtained through a coil and called the *coil-current* or the *alternating* current. Silk-covered conducting cords carry the current and end in flat metal pads called the *electrodes* or *poles*, which are either positive (anode) or negative (kathode). Instead of a flat pad a brush may be used. The negative electrodes act quicker upon a nerve or muscle than the positive, and for this reason the negative pole is applied to paralytic states, whereas the less irritable positive electrode is used for conditions with spasm or neuralgia. The battery-current is the form of electricity which is most often used in giving an electric bath. This is a most stimulating method of treatment in cases of malnutrition and general "run-down" or neurasthenia; cases of insomnia, of stuporose melancholia, and that form of insanity which is described as premature dementia in young persons. When using the electric bath—which must be of glazed earthenware to render it non-conducting—the electrodes are large metal plates, placed one at the head and the other at the foot of the bath, and separated from the patient by some non-conductor such as wood or flannel. The bath is prepared with water at 100°, and the current is then passed through it, the patient receiving about one-eighth part of the total electricity, and he remains in the bath from ten minutes to half an hour, the bath being given about three times a week. It is well to begin with a weak current, gradually increasing the strength until a contraction is produced.

In administering electricity avoid painful shocks. For the skin the electrodes should be dry, but for the muscles and deeper structures they should be moistened with water or a saline solution. It is found that the weight increases, also that the general tone and the state of the bodily nutrition improve under this treatment. The patient brightens, and the nurse obtains much credit from the application. Patients who have left the asylum cured and who have had the electric bath administered to them sometimes say that they date their convalescence from the commencement of this treatment. In some asylums, notably the Yorkshire Asylum at Wakefield, there is a most complete equipment of electrical apparatus for the treatment of the insane

### How to Become a Midwife: The Rules of the new Act Explained.

By E. L. C. Appel, M.B., B.S., B.Sc.(Lond.).

*Continued from page* 181.

### II.—RULES AND REGULATIONS FOR CERTIFIED MIDWIVES.

Contents.—Copy of the Regulations.—Case Books.—Forms for Sending for Medical Help.—Employing a Substitute.—Emergency Cases.—Certificates of Death, Still-birth, etc.—Abnormalities in Childbirth, and Disease.

The following is a copy of the 21 clauses of the regulations for midwives issued by the Central Midwives Board, and comprising "Directions to Midwives," "Duties to Patient," "Duties to Child," and "General" regulations.

### *Directions to Midwives.*

*Clause* 1.—The midwife must be scrupulously clean in every way, because the smallest particle of decomposing matter may set up puerperal fever. She must wear a dress of washable material, and over it a clean washable apron.

*Note.*—It is best to have the sleeves of the dress made so that the midwife can tuck them well up above the elbows.

A midwife who is attending a case in which there are foul-smelling discharges must not go direct to another case without first changing her dress and thoroughly cleansing and disinfecting her hands, forearms, and such appliances [Clause 2 (*a*) below] as she may have had occasion to use, and is obliged to take with her.

*Note.*—Unless the cleansing process be thoroughly carried out there will be, even after a healthy confinement, remains of blood, lochia, or liquor amnii on the fingers, and especially under the nails, which will there undergo decomposition, and so become dangerous to the next patient attended. The midwife must, therefore, keep her nails cut short, and preserve the skin of her hands as far as possible from chaps and other injuries.

*Clause* 2.—When called to a confinement a midwife must take with her:—

(*a*) An appliance for giving vaginal injections, an appliance for giving enemata, a catheter, a pair of scissors, a clinical thermometer, and a nail-brush.

(*b*) An efficient antiseptic for disinfecting the hands, etc.

(*c*) An antiseptic for douching in special cases.

(*d*) An antiseptic lubricant for smearing the fingers, catheters, douche nozzles, and enema nozzles, before they touch the patient.

*Clause* 3.—On each occasion of touching the genital organs or their neighbourhood the midwife must previously disinfect her hands and forearms.

*Clause* 4.—All instruments and other appliances brought into contact with the patient's generative organs must be properly disinfected.

*Clause* 5.—Whenever a midwife has been in attendance upon a patient suffering from puerperal fever, or from any other illness supposed to be infectious, she must disinfect herself, and all her instruments and other appliances, to the satisfaction of the local sanitary authority, and must have her clothing thoroughly disinfected before going to another

### HOW TO BECOME A MIDWIFE—*Continued.*

labour. Unless otherwise directed by the local supervising authority, all washable clothing should be boiled, and other clothing should be sent to be stoved (by the local sanitary authority), and then exposed freely to the open air for several days.

*Duties to Patient.*

*Clause* 6.—If a midwife has charge of a lying-in case she must not leave the patient after the commencement of the second stage, and she must stay with the woman until the expulsion of the afterbirth, and as long after as may be necessary. In cases where a doctor has been sent for on account of the labour being abnormal or of there being threatened danger, she must await his arrival and faithfully carry out his instructions. (See Clauses 12 and 17 below.)

*Clause* 7.—Before making the first internal examination, and always before passing a catheter, the midwife must wash the patient's external parts with soap and water, and then swab them with an antiseptic solution. For this purpose, and for washing the external parts immediately after labour and during the lying-in, on no account must ordinary sponges or flannels be used, but material which can be boiled before use and thrown away afterwards, such as linen, cotton wool, cotton waste, tow, etc.

*Clause* 8.—No more internal examinations should be made than are absolutely necessary.

*Clause* 9.—On the birth of a child which is in danger of death, the midwife shall inform one of the parents of the child's condition.

*Clause* 10.—The midwife must remove soiled linen, blood, fæces, urine, and the placenta from the neighbourhood of the patient and from the lying-in room as soon as possible after the labour, and in every case before she leaves the patient's house.

*Clause* 11.—The midwife shall be responsible for the cleanliness, and should give full directions for securing the comfort and proper dieting of the mother and child during the lying-in period, which shall be held, for the purpose of these regulations and in a normal case, to mean the time occupied by the labour and a period of 10 days thereafter. [See Clause 17 (*c*).]

*Clause* 12—A "case of normal labour" in these regulations shall mean a labour in which there are none of the conditions specified in Clause 17 below.

*Duties to Child.*

*Clause* 13.—In the case of a child being born apparently dead, the midwife should carry out the methods of resuscitation which have been taught her.

*Clause* 14.—As soon as the child's head is born, and if possible before the eyes are opened, its eyelids should be carefully cleansed with a suitable antiseptic lotion.

*General.*

*Clause* 15—No midwife shall undertake the duty of laying out the dead, or follow any occupation that is in its nature liable to be a source of infection.

*Clause* 16.—A midwife must enter in a book, with other notes of the case, all occasions on which she is under the necessity of administering any drug, whether scheduled as a poison or not, the dose, and the time and cause of its administration.

*Clause* 17.—In all cases of abortion, of illness of the patient or child, or of any abnormality occurring during pregnancy, labour, or lying-in, a midwife must decline to attend alone, and must advise that a registered medical practitioner be sent for, as, for example, under the following circumstances:—

(*a*) In the case of a pregnant woman:—

1. When she suspects a deformed pelvis.
2. When there is loss of blood.
3. When the pregnancy presents any other unusual feature (as, for example, excessive sickness, persistent headache, dimness of vision, puffiness of face and hands, difficulty in emptying the bladder, incontinence of urine, large varicose veins, rupture), or when it is complicated by fever or any other serious condition.

(*b*) In the case of a woman in labour:—

(1) In all presentations other than the uncomplicated vertex or breech; in all cases of breech presentation in primiparæ; in all cases of flooding and convulsions; and also whenever there appears to be insufficient room for the child to pass, or when a tumour is felt in any part of the mother's passages.

(2) If the midwife when the cervix has become dilated is unable to make out the presentation.

(3) If there is loss of blood in excess of what is natural, at whatever time of the labour it may occur.

(4) If an hour after the birth of the child the placenta has not been expelled, and cannot be expressed (*i.e.* pressed out), even if no bleeding has occurred.

(5) In cases of rupture of the perinæum, or other serious injury of the soft parts.

(*c*) In the case of lying-in women, and in the case of newly-born children:—

Whenever, after delivery, the progress of the woman or child is not satisfactory, but in all events upon the occurrence of the subjoined conditions in—

I.—THE MOTHER:

(1) Abdominal swelling and signs of insufficient contraction of the uterus.
(2) Foul-smelling discharges.
(3) Secondary post-partum hæmorrhage.
(4) Rigor.
(5) Rise of temperature above 100° 4′ F., with quickening of the pulse for more than 24 hours.
(6) Unusual swelling of the breasts with local tenderness or pain.

II.—THE CHILD:

(1) Injuries received during birth.
(2) Obvious malformations or deformities, not inconsistent with continued existence.
(3) Concealed malformations—incapacity to suck or take nourishment.
(4) Inflammation, to even the slightest degree, of the eyes, eyelids, and ears.
(5) Syphilitic appearance of the skin in certain parts.
(6) Illness or feebleness arising from prematurity.
(7) Malignant jaundice (icterus neonatorum).
(8) Inflammation about the umbilicus (septic infection of the cord).

(*d*) In all cases of the death of a woman during pregnancy, labour, or lying-in.

When a registered medical practitioner is sent for the midwife must state in writing the condition of the patient and the reason of the necessity for medical advice, in accordance with Clause 19 (*b*).

[These articles are published in book form by the Scientific Press, Ltd., 28 and 29 Southampton Street, Strand, W.C., at 1s., and 1s. 1d. post free. All midwife forms, price 1d. each, postage ½d. extra, can also be obtained from the Scientific Press.]

(*To be continued.*)

### Nursing in a Bergen Infirmary.

BY AN ENGLISH NURSE.

MY first visit to the Bergen General Infirmary was paid in order to see whether my desire to become a nurse was more than the common whim of a girl. I remember the doctor who obligingly offered to take me with him on one of his rounds, showing me a woman, and explaining to me —in English—how she had attempted suicide by stabbing herself, most probably to revenge herself on her husband in some matrimonial squabble. I remember, too, my tennis shoes sticking to the newly-varnished floors, and as I continued to release them alternately, I remarked to the nurse, "It seems as if I were going to stick to this hospital!" At my next visit, many years after, I could hardly believe it to be the same building. Not only had the entire staff of doctors and nurses changed, but everything had assumed a different aspect.

### THE TWO HEAD NURSES.

Instead of the black-robed deaconesses there are now young girls, most of them training for the Red Cross Nursing Society, and under the control of two head nurses—superiors —one a surgical nurse, trained in Germany, whose antiseptic talents have made her services coveted by the chief doctor at the lying-in hospital; her whole heart is in her work and her energy is untiring. The other, the medical head nurse, who was trained at the Infirmary in Edinburgh, speaks English fluently and holds a three-years' certificate. These two superiors are addressed as "Fröken" while all the other nurses are called "Sisters." Very few of them come from the educated classes, and it is no easy task to train them, though by no means a hopeless one, for several sisters have proved both good and useful members of their profession.

### THE NURSES OR SISTERS.

The Red Cross Nurses were started in Norway about ten years ago, and though their number increases every year, the demand for them is far greater than the supply. Every autumn three of the infirmary doctors give courses of lectures on anatomy, nursing, etc. Ladies who do not intend to be nurses often attend these lectures, while those who desire to go in for training, help at the same time in the wards, and thus test their practical efficiency. If a would-be nurse gives satisfaction and passes her examination, she is admitted into the hospital and put in charge of one or two wards, according to their size. Here her real two years' training begins with practical surgical and medical work, and lectures two evenings in the week during the winter. The nurses go on duty at 6 A.M. They have each to get their wards straight by 8.30 when the doctors' visits are expected; charwomen come from the town and wash the floors of the wards and passages with a wet cloth. Operations begin at 8 A.M. This hour seemed remarkably early to me at first, but it fits in better with the private work of the doctors, and they would rather risk tumbling over the bucket of a belated charwoman than alter the time.

### THE PATIENTS.

There are 136 beds in the hospital, no wards containing more than 10 beds. If any heavy case needs two nurses the superior sister always assists, or where the younger sister is inexperienced the superior sister carries out the doctor's orders. Each patient has an electric bell, so that the frequent absence of the nurse from the ward is not of serious consequence, since a patient who is incapable of ringing can always get one of the other patients to do it for him. Critical cases, especially abdominal operations, have extra nurses, or rather reliable women from the town, who, though untrained, are engaged for this purpose. Those patients who are up and about are expected to help their ward sister and wait on each other. This breaks the monotony of the day and takes them out of themselves. If a patient exhibits dirty habits the doctor neither spares him, nor scolds the nurse. A broken leg is not accepted as an excuse, and the patient will return to his farm the better for a timely correction. The nurse is not, of course, exempted from any blame which she may deserve. If she gives a report in a muddling or misguiding way she is told of it in terms that she is not likely to forget in a hurry.

### THE BUILDING ITSELF.

This is the oldest hospital in the country. A pest-house was established a century earlier in Christiania, when the plague raged there; but not until Dr. Erichsen's time—the Bergen town physician—was there anything of the kind here. He first lectured on anatomy to the town surgeons; and buying a house that had been used as a tobacco factory, he turned it into a hospital in 1754. After his death in 1768 it fell into disuse, and it was 24 years later when the parish Guardians rebuilt the hospital with some form of order. There was an asylum next to the hospital, of the kind well known all over Europe at that time, when humanity to the insane was considered superfluous. When the present asylum in Sandvigen was built some 20 years ago, the old asylum was bought by the Hospital Committee, and now only a few deserted cells, used for storage, bear witness to the suffering of the insane in old days. I happened to use the word "cell" for some other half-underground storage places, when the Governor was showing me round, and was corrected by him for not calling them "rooms." He told me that in his time, before the Board of Health became so fastidious, they were used for ordinary cases. The hospital has received very few legacies and donations; this may be the cause of the expenditure being necessarily more in the direction of modern appliances than in any striking decorations. Doctors, rather than inexperienced Guardians, have the upper hand, and though the building is old and inconvenient, it is remarkable how well it has kept up with the times—the fact that a new hospital is being built in a more suitable site, has not been an excuse for slackness on the part of the authorities.

### PHOTOGRAPHY TO THE FORE.

Under the kitchen and laundry, which form one building, there is an engine-room, where a skilled engineer is responsible for the lighting by electricity of the house and garden. The cytoscope is also lit by electricity. The hospital keeps an apparatus for Roentgen rays, both for treatment of patients and for photography. The head nurse takes any ordinary photographs the doctors may wish to have. One patient amused me by his disappointment at his face not having been included in the photograph, taken for a collection of hearts, upon which the doctor was going to lecture at some medical meeting. Cases for operation are put into a separate building, fitted with wards, bathrooms, etc., like the ordinary isolation hospitals, but with the addition of a theatre in the centre of the building. Fractures are also treated here, as the beds and entrances to the wards are more fitted for them than the ordinary beds and staircases of the old block. Septic cases are confined strictly to the old wards, and the old theatre is set aside for minor operations and septic dressings. It is always kept warm and ready for any casualty that may come in. Fre-

### NURSING IN A BERGEN INFIRMARY—*Continued.*

quently seasons of great festivity are marked by some young fellow from the country being brought in seriously damaged —the result of carelessness while celebrating some occasion with dynamite. I know one man who lost both his hands in that way, and another his eyesight.

### Absence of Old Chronic Cases.

Passing through the medical wards, one is struck by the absence of "grannies" and "daddies"—only to be accounted for, I believe, by the number of homes for the old and infirm in Bergen; while as for the sick old people in the country, "Well, they must die some day, and prefer to be in the old homestead." Several beds are as a rule filled by servant girls from the town, their mistresses having insured them against a certain number of weeks' illness. Delirium tremens cases are very rare. They, and noisy cases likely to disturb the other patients, are put in an outhouse where the men-servants sleep.

### Treatment of Phthisis.

Owing to the damp climate of the western coast of Norway, there are generally some interesting cases of nephritis, which involve a considerable amount of laboratory work in the hospital. All lung cases are treated with the open-air cure. In a third story of the building there are two wards reserved for consumptive patients. That for the women is very attractive, being sunny, and commanding a splendid view of the shipping docks and Fjord. The men wear red caps during the winter, which give a most amusing impression in their ward. Two dark-eyed pretty little fellows made ideal national "Yule Nisse" (Christmas Trolds) in their caps. Each bed is covered with a clean sheet, renewed every day for fear of infection. A metal fan to hold before their mouth when they cough and lay over the spittoon is provided for each of them. There are still two special rooms on this floor for private patients, who can likewise be received in the wards of more modern date. There are more than 60 doctors in Bergen, where the population is about 77,000. Any doctor may send his patients to the hospital, but in doing so he resigns all further treatment of them; though where the disease is of professional interest, he is welcome to watch its development.

### Chief Surgeon and Physician.

The chief surgeon resides with his family in the main building. He superintends the whole establishment, but a guardian and a secretary assist him in his most irksome duties. He goes abroad every year to Paris or Berlin. Next to him is the assistant or "reserve" surgeon, with the corresponding "reserve" physician, who are newly appointed every three years, in order to enable a larger number of doctors to profit by the varied experiences of these posts. They live out in the town, and their hospital duties do not prevent them having a private practice. They make two rounds of the wards during the day, and are telephoned for should any patient come in requiring immediate attention. The salary is small, but the professional advantages are so decided as always to ensure a fair number of applicants for each vacancy. The "house" surgeon's and "house" physician's offices are only held for six months. They are generally filled by medical students, who may or may not have passed their final M.B. exams. They take it in turns to be on duty, and attend to any trifling casualty that comes in.

### Infectious and Lock Wards.

One wing of the hospital is shut off for infectious cases. Typhoid is considered as such, and two wards are set apart for that. Wet-packing to keep down the temperature is practised more than the German method of baths. Diphtheria has another ward. During an epidemic in the town, three cases requiring tracheotomy were admitted in one week. In the same wing, a story higher, there is a lock ward for women. Patients are sometimes allowed to go out in the town during their stay in the hospital, and are given tickets stating the time they are to be back, etc. Should they abuse their freedom and return intoxicated, they are either discharged or punished by having to spend a couple of days in bed, and the disgrace is duly impressed on them.

### The Pride of the Infirmary.

The pride of the Infirmary is the bath-house built by the late "Overlæge" Jens A. Holmboe in 1875. His statue is placed at the entrance of the private bath-rooms. Steam, fresh, salt, mud, sulphur, fir-needle, dry-heat, and other baths can be obtained here. The head bathing man and woman are trained masseurs. The town midwives are given free admittance. Cheaper and commoner baths are also provided in the town for the very poor. It is to be hoped that before long these public baths may be introduced on a small scale into each country parish, so that the old custom of an annual Christmas bath (which is fast dying out) may be promptly superseded by something better. At present in some fjords it looks as if the only experience of the kind known by many of the peasants has been at the hands of the parish midwife at their birth—an historic event in many cases! Those of the patients who can stand it are carried by the porters in a chair, well wrapped up, to the bath-house. I should much prefer bathing my own patients to trusting them to the bath attendants, especially in the case of children, where it is so important that they should regard a tub as the great treat of the day. We did it once for three little tubercular sisters, having a big bath carried into the ward; their mother, also tubercular, watching from her bed, remarked: "Little Ingeborg used always to scream over her bath at home, but now you can hardly get her to leave it!"

### Victoria Hospital Bazaar.

The three days' bazaar held at the Albert Hall last week in aid of the funds of the Victoria Hospital for Children was a great success, about £8,000 being realised. The amount for which a special appeal had been issued was £13,000, this being the balance of the £40,000 which is the total cost of building which is practically a new hospital, with the additional cost of furnishing and reconstructing the old building for purposes of administration, and providing accommodation for resident medical officers and extra nursing staff. The new wing consists of six wards, entirely self-contained, an isolation department with eight beds, an operating theatre, and an *x*-ray department. The additional wards increase the accommodation from 42 to 104 beds, but two wards, containing 32 beds, are closed until sufficient financial support is forthcoming to justify the committee in adding to the annual expenditure, which already greatly exceeds the annual income.

The bazaar, which contained many original features, was appropriately arranged to represent old nursery favourites, each stall having a sign in some way connected with the wares offered for sale; while six stalls, occupying a central position, were crowned with large figures representing well-known nursery rhymes. The hospital stall, under the care of the matron, sisters, and nurses of the hospital, was effectively set out with miscellaneous wares, many of them made by the staff. The Queen, who attended the bazaar on the first day, made a large number of purchases. The sale terminated at six o'clock on Thursday, when a swift transformation was effected, and a ball, at which many distinguished persons were present, concluded this special effort on behalf of the sick children.

### Everybody's Opinion.

[Correspondence on all subjects is invited, but we cannot in any way be responsible for the opinions expressed by our correspondents. No communication can be entertained if the name and address of the correspondent are not given as a guarantee of good faith, but not necessarily for publication. All correspondents should write on one side of the paper only.]

### A GRIEVANCE AT STAPLETON, BRISTOL.

"P. S." writes: We rarely take up a paper but we see something about phthisis, and what is being done to endeavour to cure it in various ways. The Guardians of the Workhouse Infirmaries at Bristol are apparently trying to do just the reverse. The Infirmary itself is not overmodern, far different from the new small workhouse infirmaries, which are models. There are no diversions for the nurses, but "work"; and now a new grievance is added. At the back of the nurses' home, on the only piece of garden, the Guardians are building huts for phthisical women, within a few feet of the nurses' sleeping apartments. So there may now be a few more added to the overcrowded number of patients with this disease.

### A QUESTION OF RELIGION.

"CONVERT" writes: I am sure there are many nurses as well as myself who will feel grateful to Father Fitzpatrick for his letter on the above subject. Not only in Nice, but in many institutes in England, Catholic nurses have experienced the same tyranny; either the authorities will not engage them at all, or, if they were formerly Protestants, they are asked to resign, quite oblivious of the fact that the nurse has served faithfully hitherto. Speaking for myself after I had served faithfully for nearly three years, when I mentioned my changed views, a special committee was called and I was told to resign. Also last summer, I had only temporary work from the same cause. I have often noticed that a nurse belonging to any other religion, though she may never enter a place of worship of her own free will, is tolerated. I have never been able to see how any man or woman can live without religion. The majority of Protestant nurses know full well how many times they are thrown among people whose ways and surroundings are, to say the least, decidedly risky, and, unless we can feel in close touch with a Higher Hand, or go where we can be constantly reminded of our duty in the highest sense of the word we stand a good chance of drifting to a sad destiny. Consequently, as sane women, may we not please ourselves and choose our own faith? I see that Mr. O'Brien has gained a point for King's College nurses Will not some one try for the same tolerance for all the others?

### Appointments.

[No charge is made for announcements under this head, and we are always glad to receive, and publish, appointments. The information, to insure accuracy, should be sent from the nurses themselves, and we cannot undertake to correct official announcements which may happen to be inaccurate. It is essential that in all cases the school of training should be given.]

AYR DISTRICT ASYLUM.—Miss Gertrude Binning has been appointed assistant matron. She was trained at the Western Infirmary, Glasgow.

HAM GREEN ISOLATION HOSPITAL, BRISTOL. — Miss Caroline Golledge has been appointed assistant matron. She was trained at Crumpsall Infirmary, Manchester, and has since been sister at Queen Charlotte's Hospital, London, charge nurse at Ham Green Hospital, and sister at Crumpsall Infirmary. She has also done private nursing at Clifton.

NOVERS HILL HOSPITAL, BRISTOL.—Miss Annie Watt has been appointed matron. She was trained at Middle Ward Hospital, Motherwell, and the Western Infirmary, Glasgow; and has since been sister-in-charge of the Small-pox Hospital, Bellshill, Lanark. She has also been nurse at the Fever Hospital, Peterhead, Aberdeenshire.

PLAISTOW FEVER HOSPITAL.—Miss Jessie E. Ives has been appointed assistant matron. She was trained at Plaistow Fever Hospital, and the London Hospital, Whitechapel, and has since been sister at Plaistow Fever Hospital.

POPLAR AND STEPNEY SICK ASYLUM.—Miss Kate Florence Keeping has been appointed second assistant matron. She was trained at Southwark Infirmary, East Dulwich, and has since been housekeeper at the Western Hospital, Fulham, and ward sister and night superintendent at the Poplar and Stepney Sick Asylum.

RAMSGATE GENERAL HOSPITAL.—Miss Lillian Fincke has been appointed sister. She was trained at the Royal Albert Hospital, Devonport, and has since been sister at North Devon Infirmary, Barnstaple.

ROYAL SURREY COUNTY HOSPITAL, GUILDFORD.—Miss Margaret Traill has been appointed matron. She was trained at Guy's Hospital, London.

ROYAL VICTORIA HOSPITAL, BELFAST.—Miss Maude McClelland has been appointed staff nurse. She was trained at the General Hospital and Dispensary, Rotherham.

WORKHOUSE INFIRMARY, WITTINGTON, MANCHESTER.—Miss Emily M. C. Legat has been appointed assistant hospital matron. She was trained at the Royal Edinburgh Hospital for Sick Children and St. George's Hospital, London. She has since been night superintendent at the Royal Edinburgh Hospital for Sick Children and superintendent of the Nurses' Home, Chelsea Infirmary.

### Death in Our Ranks.

ON Wednesday last week Miss Sarah Elizabeth Hanson died at the Cottage Hospital, Brixham, from cerebral apoplexy. She was the district midwife, and lived in the hospital. At the funeral the bearers of the coffin were all Volunteers, and several of them husbands of old patients, an evidence of the respect in which Miss Hanson was held by the people.

### TRAVEL NOTES AND QUERIES.

BY OUR TRAVEL CORRESPONDENT.

PENSIONS IN BRUGES OR ST. MALO (F. M.). Have you received any answer by post to your question? I think you have been confused with another correspondent. Let me hear at once. Also when you want to take your holiday and how much you can *each* afford to spend; likewise how long is your holiday to last. If you will give me full particulars I will help you in every way I can.

HOLIDAYS IN ILFRACOMBE, ETC. (MRS. L.). We do not reply by letter unless 2s. 6d. is sent for our Convalescent Fund. It would have been more satisfactory if you had told me definitely what you could afford. "Inexperience" is rather vague. In Ilfracombe Mrs. Dyer, Belle Vue House, Portland Street, takes boarders at moderate terms, or Mrs. R. Ware, 9 Axford Park. No small hotels at low prices. At Cromer there is an excellent boarding house for nurses at very reasonable terms. Apply "The Lady Superintendent," St. Mary's. Terms from 25s. per week. At Whitby Miss Wilman, Farndale Boarding House, 5 Church Square, or Mrs. Stonehouse, 10 West Terrace.

RULES IN REGARD TO CORRESPONDENCE FOR THIS SECTION.—All questioners must use a pseudonym for publication, but the communication must also bear the writer's own name and address as well, which will be regarded as confidential. All such communications to be addressed "Travel Editor, 'Nursing Section of *The Hospital*,' 28 Southampton Street, Strand." No charge will be made for inserting and answering questions in the inquiry column, and all will be answered in rotation as space permits. If an answer by letter is required, a stamped and addressed envelope must be enclosed, together with 2s. 6d., which fee will be devoted to the objects of "The Hospital" Convalescent Fund. Any inquiries reaching the office after Monday cannot be answered in "The Hospital" of the current week.

### Echoes from the Outside World.

### The King's Visit to Germany.

THE King left London on Thursday night last week for Kiel, and arriving at his destination on Saturday afternoon, was warmly welcomed by the Emperor William. A gala dinner was given in the Royal German yacht *Hohenzollern* in the evening. The Kaiser, in proposing the health of the King, dwelt on the peaceful mission of the German fleet, and prayed that success might crown the efforts of King Edward and himself for the preservation of peace. The King, replying in German, said: "May our two flags float beside one another to the most distant times, as they float to-day, for the maintenance of peace and for the well-being, not only of our countries, but of all other nations." On Sunday morning the day of rest was observed in the *Hohenzollern*, the *Victoria and Albert*, and the ships of war, and Divine service was conducted on board. In the evening, the King entertained the Emperor and Empress, the Princes and Princesses, and a large company on board the *Victoria and Albert*. On Monday the King and Emperor visited the Government dockyards and the Germania dockyards of Herr Krupp. Their carriage passed between a double line of 1,000 schoolgirls dressed in white, who were all armed with a bunch of flowers, which they threw into the carriage, almost covering the monarchs with floral tributes. In the afternoon they witnessed a regatta of all the ships' crews, and the distribution of the Kiel Regatta prizes took place on board the *Hohenzollern* in the presence of the King. On Tuesday the King visited Hamburg and had an enthusiastic reception.

### The Queen and the Youthful Musicians.

QUEEN ALEXANDRA is always particularly anxious to encourage rising musical talent. On Monday afternoon she summoned to her presence two musical prodigies. The first was little Florizel von Reuter, a *protégé* of the Queen of Roumania, aged 12, who is well known on the Continent, has composed a symphony which he has dedicated to her Roumanian Majesty, and which is rendered by a full orchestra conducted by the youthful musician himself. He received the Royal summons on Monday, just as he was going to rehearse with the orchestra, and was obliged to request the performers to wait whilst he went to Buckingham Palace. Here he played four pieces on his violin, but when asked to have tea afterwards, to the Queen's amusement, begged to be excused, because he felt that it was his duty to hurry back to his waiting musicians. Later in the afternoon Franc von Vescey, the child violinist, of 11, who has quite taken London by storm, also performed by request of the Queen, accompanied on the piano by his mother. He has already visited the Palace by command two or three times.

### The Birthday Honours.

THE list of Honours issued on the King's birthday includes three Privy Councillors, one of whom is Mr. Charles Booth, the well-known social reformer and statistician; seven new baronets, of whom one is Mr. Alfred Harmsworth, founder of *Answers* and the *Daily Mail*; and 26 new knights, including Dr. Edward Elgar, the musical composer; Professor Dewar, the distinguished Cambridge scientist; Mr. G. S. Gibb, general manager of the North-Eastern Railway; Mr. Constantine Holman, a well-known member of the medical profession; and Dr. Stevenson, scientific analyst to the Home Office. Among other honours conferred, the Kaisar-i-Hind medal of the first class was bestowed upon Miss E. A. Manning, secretary of the National Indian Association. No fewer than 318 persons, men and women, have been given the Imperial Service Medal. This list includes postmen, prison warders, telegraphists, employés in the Royal Dockyards, and messengers in Government Offices.

### The War in the Far East.

GENERAL KUROPATKIN has taken the command of the Russian Army at Liau-yang in person. In a despatch to the Tsar, he says that the Japanese Army advancing from Kinchau is gradually carrying out its northward movement. The advance of General Kuroki's army from Sin-yen is suspended, obviously with the object of bringing the advance guards of the two armies into line. The Japanese, he adds, are throwing up field fortifications on the road from Sin-yen to Kai-chau, and also at a point north-east of Saimatse.—Admiral Togo reports from Tokyo that on Thursday, the 23rd, the Russian fleet left Port Arthur and anchored for the night in the outer roadstead. During the night the Admiral, having been informed of their movements by wireless telegraphy, attacked the enemy. A battleship was sunk in six fathoms, and a string of lame ducks limped back into the inner harbour the following day.—An official telegram received at the Japanese Legation says that one battleship, *Sevastopol* type, and one cruiser, *Diana* type, were towed into Port Arthur apparently seriously damaged. A Japanese destroyer and three torpedo boats were slightly damaged. The fleet re-entered Port Arthur on Friday and has not since appeared.

### A Music Loan Exhibition.

A SPECIAL loan exhibition of musical instruments was opened at the Fishmongers' Hall, London Bridge, on Monday by the Prince of Wales, who was accompanied by the Princess. The exhibition has been organised by the Worshipful Company of Musicians in commemoration of the tercentenary of the granting by King James I. of the Company's second charter of incorporation, and will remain open for three weeks. During this time Sir Frederick Bridge will daily give lectures, illustrated by music for sackbuts and cornets, and by selections published in 1599, which includes Shakespeare's "O Mistress Mine" with the six instruments for which the music was originally written, the lute, the cittern, the pandora, the treble viol, the bass viol, and the recorder. The Royal visitors were much interested in this portion of the programme and in the Handel and the Purcell relics. The collection of instruments, pictures, books, and MSS. is the most complete yet seen in England, and is of priceless value. The King lends several MSS. from the Buckingham Palace library, including Handel's autograph score of "The Messiah."

### "Living Bridge" Fête.

THE living game of "Bridge," which was played at Hengler's Circus on Monday night, after the manner of "living" whist, attracted so much attention, that the example is likely to be followed by others in need of a novel entertainment to help the funds of a charity. The game was arranged at the initiative of Lady Bective and others, and was designed to aid the St. Helena Hospital Home, which provides accommodation for girls from 16 to 26 years of age; and Princess Christian, who is President, was present with her daughter, Princess Louise. The circus was beautifully decorated, and coloured lights, which changed from time to time, made the scene still more effective. The players in the game were all well-known Bridge players, and they stood in bowers at opposite points in the circumference of the arena. The 52 living cards wore handsome fancy dresses, the hearts in scarlet with silver capes, the diamonds in scarlet and white, the clubs in yellow and black, and the spades in black and gold. All the ladies had powdered hair and wore large black Gainsborough hats, and the men had Watteau costumes.

### A Book and its Story.

### REMINISCENCES OF BOHEMIAN LIFE IN PARIS.

In her new book of reminiscences Miss Corkran recalls early days, when as an Art student in Paris she led the gay Bohemian life associated with the idea of study in the Ateliers. There are also anecdotes of some celebrities omitted in her previous book "Celebrities and I," and many others of "Oddities." The chief interest to the reader, and the characters about whom it has given her most pleasure to write are those persons, classed as "delightful failures," who, through a too sensitive organisation and an unselfish nature, have found the battle of life too hard for them, and so, to quote the authoress, "have had to take a back seat in the world's great show." But the brighter side of life dominates the book on the whole. It is addressed "to those unconventional genial souls who see life through kindly spectacles, and who have the sense of fun highly developed."

Among the so-called failures was one who was at the same time a real artist, a M. Poulain, whom Miss Corkran met in Paris. "He is too original, said Dr. C., and too independent, and keeps away from society; he does not make money. He has executed some enchanting pictures of Paris and its environs. He has a charming daughter, who is the type of the Parisienne—*mutine*, with nice manners, and pretty gestures." Miss Corkran and her friend, Dr. C., were on their way to call on M. Poulain in Quartier Luxembourg. When they arrived at the entrance to the courtyard in which the house was situated, they saw the artist in his shirt sleeves filling a pail of water from a pump, which he explained by saying, "You see I am helping my *femme de ménage*; she is rather rheumatic, so I fetch the water while she peels the vegetables." It was the fête of the artist who was 68 on that day. As a specially good dinner was being prepared for which a sister in the country had sent a poulet, fish, and grapes and peaches, his visitors accepted a pressing invitation to dine. The description of the pretty daughter Mimi is essentially that of a young Parisienne. "We went up four flights of steps; on the landing stood a young girl. She had a charming face, brown radiant eyes, retroussé nose, olive complexion, rather a large smiling mouth, showing pretty, even white teeth, thick coils of red-brown hair twisted round her head, and extremely small arched feet in dainty high heeled shoes." They were ushered into the atelier of M. Poulain in which artistic disorder prevailed. A big cobweb stretched across the ceiling of the studio. "I never allow any one to disturb my spiders," said M. Poulain, looking affectionately at a long-legged spider crawling on the web. "Spiders are to me living sermons. I envy their skill, patience, and power of work. This web is a triumph. I don't allow Mimi, or the servant, or anyone, to dust or clean this place, except myself." Though there was a charcoal smudge across his cheek, and his appearance was generally in keeping with his studio, the face was so full of intelligence, and his eyes shone with a fire and depth that made his visitors oblivious to everything but his interesting personality. In pulling out a large canvas for their inspection he disturbed a fat old dog, who was asleep behind it. "I cannot live without animals," he exclaimed, "they are so true, and faithful, and interesting." The canvas showed a large, dreamy landscape, an evening scene near a river. The artist was a disciple of Corot's. He was delighted when Miss Corkran noticed this. "Ah! you have done me good," he exclaimed, seizing both my hands and squeezing them. "He is the painter *par excellence* whom I adore and venerate. There is no one like Corot. Ah, if I remind you of his work, I die happy. What a poet! There never has been, and there never will be again such an exquisite and original painter. He is unique. Ah, why did *le bon Dieu* take him away? Why are not geniuses allowed to live longer than the médiocres? I intend when I meet *le bon Dieu* to ask him several questions which He alone can answer. There are troops of nonentities who live on till over 80 years of age. Corot loved Nature. I have seen him in ectasy over fields, flowers, foliage, sunrises, sunsets. *Oh, ce cher Père Corot*, so simple, so gifted, so generous. If any of we poor *rapins* wanted money we could help ourselves. He had a bowl filled with pieces, and if any of his friends were in want we took what we really wanted. No one abused the privilege, because we were all touched and deeply grateful." When dinner was over, M. l'Abbé Dubois was announced. The Abbé projected an atmosphere of peace and goodwill. He was nearly 80. Life had certainly imprinted its marks on the lines and features of his face. The record of the Abbé was a noble one. A holy, inner light shone out from his tender eyes, from his sensitive mouth. The forehead was broad; the white hair fell on his neck. His appearance was really saintly; it made one feel that the spirit lives after the body is dead. His life was the best of sermons. "He understands pities, woes—that is the secret of his influence," as Mimi said. When sketching at Berck-sur-Mer, Mr. W. G. Wills, the dramatist, was encountered. "This delightful oddity wore a heavy ulster coat (it was an August day), in the hood of which he carried a paint-box, brushes, and a bottle of turpentine and oil. His feet were bare, on his head he had a straw helmet, the worse for wear; an urchin in rags was carrying a sketching-easel and canvas." After some interchange of views on art, Mr. Wills exclaimed, "Come along with me while I put some sea in my picture. It must be a blue sea; to-day is like a big turquoise." It was a scene that Turner might have done justice to, but Mr. Wills certainly was unable to cope with it; he did not attempt to copy what was before him. He squeezed out a wonderful mixture of colours—then taking a knife he spread it like butter on his canvas. "When this dries I shall glaze on transparent tints; it will then be a dream of exquisite colouring—the sort of light that comes through a cathedral window. Nature often has attacks of scarlet fever and measles. It is not advisable to be too literal in one's interpretations." Miss Corkran adds, "What a contrast between M. Poulain's and Mr. Wills's attitude. The former always approached nature with the faith of a little child. He would have considered Mr. Wills's art as a preposterous impertinence. Mr. Wills was on the wrong track, carried away by imagination. At all events, he was happy, much more so than when he wrote plays for Sir Henry Irving, and received fine payment for his work." A chapter on Rye and the artists' colony there, with interesting notes of that quaint little place, and some genial recollections of Mr. Henry James, who lives in "Lamb House," an old mansion linked with a bygone world, haunted by memories of Mary Wortley, Lady Suffolk, and Queen Caroline, closes a book in which the authoress owns that precision in the narration of a good story is not vital to its purpose, if to excite amusement is its *raison d'être*. Therefore, we take the book and its anecdotes as she intends them to be received, "as a record of impressions rather than accurate facts."

* Oddities, Others, and I. By Henriette Corkran. (Hutchinsons. 16s.)

### Notes and Queries.

**FOR REGULATIONS SEE PAGE 143.**

*Hospital Training.*

(94) With reference to the reply to *M. B. H.* in our issue of June 18th, the Secretary of the Sussex County Hospital, Brighton, writes that probationers are now received at that institution for a three years' course of training for £15 instead of £30, £10 being paid the first year, and £5 the second.

(101) Will you kindly tell me where I could be received as probationer at 35 years of age. I am willing to pay a premium if necessary?—*E. E. L.* and *M. J.*

There are several hospitals which receive probationers up to 35 years of age. Consult "The Nursing Profession: How and Where to Train."

Can you tell me where probationers are received as young as 18? *L. H.*

See "The Nursing Profession: How and Where to Train."

Will you kindly tell me if there is in London any home or hospital for children where a young lady could be trained for two or three months?—*Brondesbury.*

Write to the Norland Institute, 10 Pembridge Square, W., or Miss Buddulph Pinchard, Court Royal, South Norwood Hill, S.E.

*Ringworm.*

(102) Some months ago I carried to and fro a small child suffering from ringworm, and shortly after a bald patch appeared on my head. I consulted a doctor who pronounced it alopecia, certainly not ringworm. After using some ointment the hair grew quickly. Surely there must be some connection between the bald patch and the ringworm?—*M. S.*

Presumably there is, but it is still obscure.

*Home.*

(103) Will you kindly tell me of a small home or asylum where a patient could be taken for one or two months' change of air?—*Nurse R.*

The Secretary, the After-care Association for Poor Persons Discharged Cured from Asylums, Church House, Dean's Yard, Westminster, S.W., would possibly give you the name of a suitable home.

Will you kindly tell me of any nursing home at the sea, or in the country, where a man could be received with a subscriber's letter? He has had a long serious breakdown through overwork, and is without means. The doctor orders him proper nourishment, cheerful surroundings under medical supervision.—*F. C.*

St. Andrew's Hospital for Convalescents and Incurables, Clewer, Windsor; the Beau Site Convalescent Home, Hastings; and if medical supervision can be dispensed with, the Merchant Taylors Company's Convalescent Home for Men at Bognor. Write for particulars to the Secretary, the Hall, Threadneedle Street, E.C. See Burdett's "Hospitals and Charities" for others.

*Patents.*

(104) I have invented a urinal of which I send you particulars and drawing. Will you kindly tell me how I could bring it before manufacturers, as I should like to make a little money by my invention and I am not in a position to bring it out myself?—*Ura.*

The invention seems good, and the drawing, etc., was returned as you requested. Write to the Patent Office, 25 Southampton Buildings, W.C., and patent your invention; then send the particulars to the various firms manufacturing medical and nursing requisites.

Be good enough to tell me where I can get an invention patented.—*M. E. F.*

See reply to *Ura.*

**Important Nursing Textbooks.**

"Nursing Profession: How and Where to Train." 2s. net; 2s. 4d. post free.

"Nurses' Dictionary of Medical Terms and Nursing Treatment." By Honnor Morten. 2s. post free.

"Nursing." By Isabel Hampton. 7s. 6d. net; 7s. 10d. post free.

"Surgical Ward-Work and Nursing." By Dr. Alex. Miles. 3s. 6d. net; 3s. 10d. post free.

"Outlines of Insanity." By F. H. Walmsley, M.D. Popular Edition. 1s. 6d. post free.

"Notes on Pharmacy and Dispensing for Nurses." By C. J. S. Thomson. 1s. post free.

The above works are obtainable of all booksellers, or direct from The Scientific Press, Limited, 28 & 29 Southampton Street, Strand, London, W.C.

### For Reading to the Sick.

"WATCH WITH ME IN MY LONELINESS."

DRAW my weary heart away
From this gloom and strife,
And these fever pains allay
With the dew of life;
Thou canst calm the troubled mind,
Thou its dread canst still;
Teach me to be all resigned
To my Father's will.

Then if I must wake and weep
All the long night through,
Thou the watch with me wilt keep,
Friend and Guardian true;
In the darkness Thou wilt speak
Lovingly with me,
Though my heart may vainly seek
Words to breathe to Thee.

Wheresoe'er my couch is made,
In Thy hands I lie;
And to Thee alone for aid
Turns my restless eye:
Let my prayer grow weary never,
Strengthen Thou th' oppressed,
In Thy shadow, Lord, for ever
Let me gently rest.

*From "The Inner Life."*

"More things are wrought by prayer
Than this world dreams of."

*Tennyson.*

Prayer is the voice of a supernatural hope, and by prayer that hope is nourished and enlarged. Prayer implies energy and eagerness in the one who prays, and a willing mind and a loving heart in the One to Whom the prayer is addressed. It is the voice of a real need; it is conditioned by the character and the revelation of God. If it is made in faith and hope—that is, if it is real prayer—it will never be unanswered, although, as God sees farther than we see, the answer may come in an unexpected form. Prayer is the very lifebreath of the soul. As the movement of the lungs is necessary for the life of the body to make use of the air we breathe, so prayer is needful for the soul that it may breathe the air of Eternity. If we will, we can cast all our care upon God, and carry to Him every anxiety, every pleasure, every sorrow, with the confidence that however long He may appear to keep us waiting, or however different the answer may seem to be from that hoped, still an answer there will be, and a good one, from Him Who is all goodness. The night may be dark, and the clouds may be gathering; life may indeed be, nay it must be, "labour and sorrow," but there is sunlight beyond the darkness, and there is rest beyond the toil.—*Canon Knox Little.*

Heavenly Father, in weakness of body and mind, again I commend my sinful self into Thy hands. Pardon me all that is past; wash me in my Redeemer's blood; clothe me in His righteousness; and accept me for His sake. Sanctify me wholly, and make me all Thine own, through Jesus Christ our Lord.—*Amen.*

